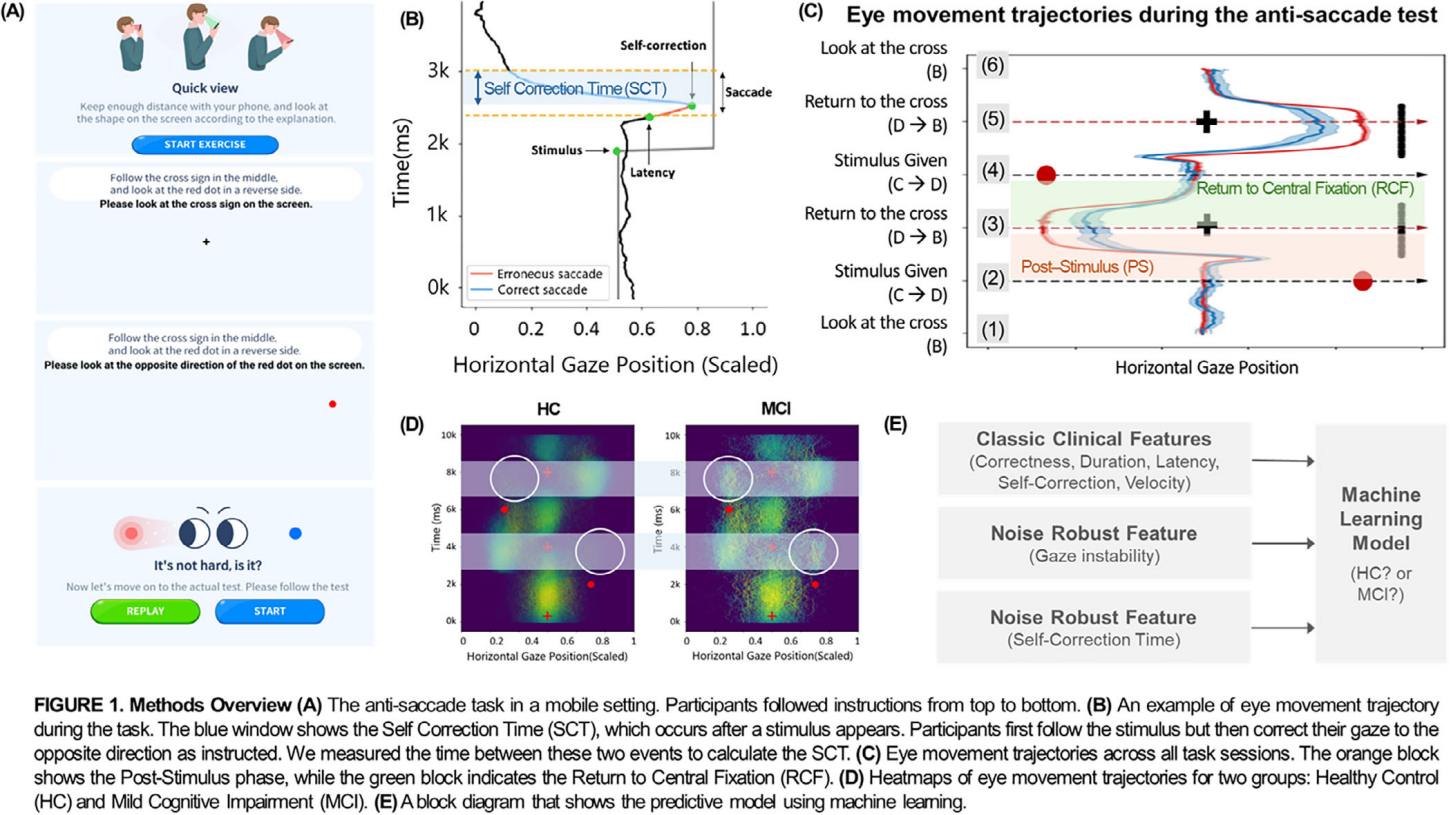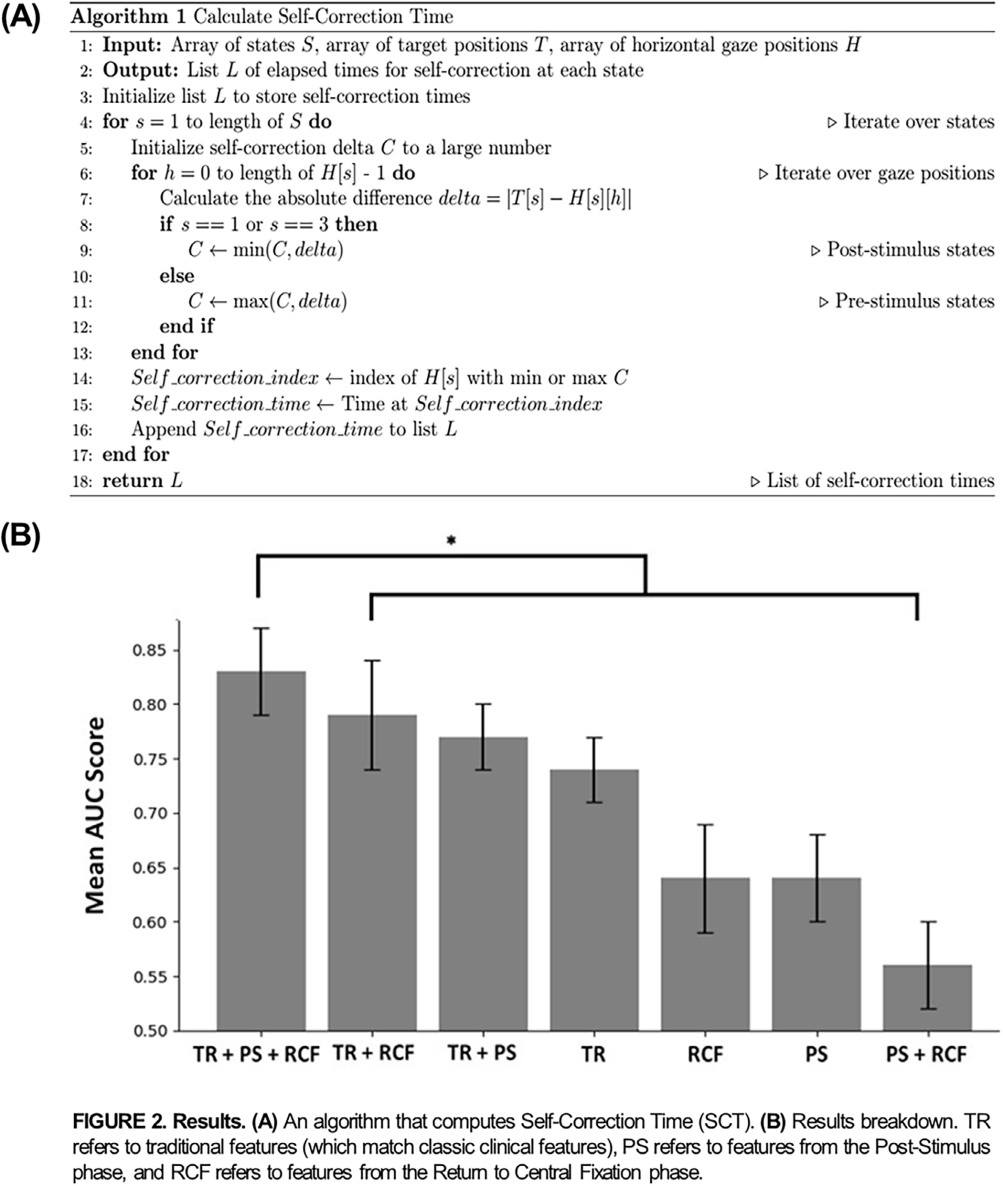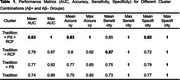# From Prodromal to Preclinical: Detecting Amyloid‐Beta Through Mobile Gaze Analysis

**DOI:** 10.1002/alz70856_102761

**Published:** 2025-12-24

**Authors:** Jin Sung Kim, Whani Kim, Hyun Jeong Ko, Byung Hun Yun, So Yoon Park, Bo Ri Kim, Jee Hang Lee, Geon Ha Kim, Jinwoo Kim

**Affiliations:** ^1^ Sangmyung University, Seoul, Jongno‐gu, Korea, Republic of (South); ^2^ HAII Inc., Seoul, Korea, Republic of (South); ^3^ Ewha Womans University, Seoul, Korea, Republic of (South); ^4^ Graduate School of AI and Informatics, Sangmyung University, Seoul, Korea, Republic of (South); ^5^ Ewha Medical Research Institute, Ewha Womans University, Seoul, Korea, Republic of (South)

## Abstract

**Background:**

The accumulation of amyloid‐beta (Aβ) characterises Alzheimer's disease (AD) functioning as an essential biomarker for diagnosis and progression monitoring. Positron Emission Tomography (PET) imaging represents an established method for this. Yet its considerable expense and invasive procedures restrict widespread clinical implementation (Jack et al., 2018). Eye movement analysis offers a practical solution to that end (Molitor et al., 2015). The distinction between Aβ⁺ and Aβ^−^ through eye movement analysis presents technical obstacles, particularly as previous investigations concentrated on Post‐Stimulus (PS).

**Method:**

The investigation examined 193 individuals aged 50 and above, with PET imaging results determining their classification into Aβ+ or Aβ– groups by neurologists. The participants completed anti‐saccade tasks through an Android eye‐tracking application, which recorded gaze data via the smartphone front camera. The analysis incorporated Classic Clinical Features (correctness, duration, latency, self‐correction, velocity) alongside two newly developed noise‐robust parameters comprising Self‐Correction Time (SCT; Figure 1A), and Gaze Instability, defined by –Mean(G)/SD(G). Previous self‐correction measurements concentrated exclusively on immediate corrections following erroneous saccades. We here redefined self‐correction as SCT, a time‐based measurement examiming intervals between incorrect saccades and their corrections (Figure 1B). The methodology incorporated the Return to Central Fixation (RCF) phase to examine post‐stimulus gaze patterns, as Aβ⁺ and Aβ^−^ groups exhibited distinctions (Figure 1C, D). We conducted decoding analysis through a machine learning model to evaluate their impact, employing assessment criteria that included area under the ROC curve (AUC), accuracy, sensitivity, and specificity (Figure 1E).

**Result:**

When incorporating all features (Classic + PS + RCF), the CatBoost model attained the best classification performance: mean AUC 0.83 (sensitivity 0.85, specificity 0.82; Table 1). Paired *t*‐tests indicated significant differences in AUC across feature combinations (Figure 2 B).

**Conclusion:**

SCT and Gaze Instability measurements demonstrated effective differentiation in mobile contexts. The redefined self‐correction parameters and Gaze Instability metrics, combined with the RCF phase, enhanced measurement precision under ambient noise. These methodological advances established an economical approach for amyloid pathology detection. Enabling potential applications for earlier Aβ identification at preclinical AD stages, it presented opportunities for expedited clinical intervention and refined disease management protocols.